# Individualized hemodynamic optimization guided by indirect measurement of the respiratory exchange ratio in major surgery: study protocol for a randomized controlled trial (the OPHIQUE study)

**DOI:** 10.1186/s13063-020-04879-x

**Published:** 2020-11-23

**Authors:** Stéphane Bar, Pierre Boivin, Younes El Amine, Richard Descamps, Mouhamed Moussa, Osama Abou Arab, Marc-Olivier Fischer, Hervé Dupont, Emmanuel Lorne, Pierre-Grégoire Guinot

**Affiliations:** 1 Anesthesiology and Critical Care Department, Amiens University Medical Center, Rond-point du Professeur Christian Cabrol, F-80000 Amiens, France; 2 Anesthesiology and Critical Care Department, Valenciennes General Medical Center, Avenue Désandrouins, F-59322 Valenciennes, France; 3 Anesthesiology and Critical Care Department, Normandie University Medical Center, UNICAEN, F-14000 Caen, France; 4 Anesthesiology and Critical Care Department, Lille University Medical Center, Oscar Lambret, F-59037 Lille, France; 5 Anesthesiology and Critical Care Department, Dijon University Medical Center, 2 Bd Maréchal de Lattre de Tassigny, F-21000 Dijon, France

**Keywords:** Respiratory exchange ratio, Postoperative complications, Major surgery

## Abstract

**Background:**

Observational studies have suggested that a high respiratory exchange ratio (RER) is associated with the occurrence of postoperative complications. The study’s primary objective is to demonstrate that the incidence of postoperative complications is lower in an interventional group (patients monitored using a hemodynamic algorithm that incorporates the RER) than in a control group (treated according to standard practice).

**Methods:**

We shall perform a prospective, multicenter, randomized, open-label, superiority trial of consecutive patients undergoing major noncardiac surgery (i.e., abdominal, vascular, and orthopedic surgery). The control group will be treated according to the current guidelines on standard hemodynamic care. The interventional group will be treated according to an algorithm based on the RER. The primary outcome will be the occurrence of at least one complication in the 7 days following surgery. The secondary outcomes will be the length of hospital stay, the total number of complications per patient, the 30-day mortality, the total intraoperative volume of fluids administered, and the Sequential [sepsis-related] Organ Failure Assessment (SOFA) score and laboratory data measured on postoperative days 1, 2, and 7. A total of 350 patients will be included.

**Discussion:**

In the operating theater, the RER is potentially a continuously available, easy-to-read, indirect marker of tissue hypoperfusion and postoperative complications. If the RER does predict the occurrence of tissue hypoperfusion, it will help the physician to provide personalized hemodynamic management and limit the side effects associated with excessive hemodynamic optimization (volume overload, vasoconstriction, etc.).

**Trial registration:**

ClinicalTrials.gov NCT03852147. Registered on February 25, 2019

**Supplementary information:**

The online version contains supplementary material available at 10.1186/s13063-020-04879-x.

## Administrative information

The order of the items has been modified to group similar items (see http://www.equator-network.org/reporting-guidelines/spirit-2013-statement-defining-standard-protocol-items-for-clinical-trials/).

**Table:** 

Title {1}	Individualized hemodynamic optimization guided by indirect measurement of the respiratory exchange ratio in major surgery: study protocol for a randomized controlled trial (the OPHIQUE trial)
Trial registration {2a and 2b}.	ClinicalTrials.gov identifier: NCT03852147. Registered on February 25, 2019. All items from the WHO Trial Registration Data Set can be found in Additional file 1.
Protocol version {3}	Protocol version 1.2, dated May 3, 2018
Funding {4}	The trial was funded by Programme Hospitalier de Recherche Clinique (PHRC) Inter-régional GIRCI Nord-Ouest 2017 (API17-03).
Author details {5a}	^1^ Anesthesiology and Critical Care Department, Amiens University Medical Center, Rond-point du Professeur Christian Cabrol, F-80000 Amiens, France. ^2^ Anesthesiology and Critical Care Department, Valenciennes General Hospital, Avenue Désandrouins, F-59322 Valenciennes, France. ^3^ Anesthesiology and Critical Care Department, Normandie University Medical Center, UNICAEN, F-14000 Caen, France. ^4^ Anesthesiology and Critical Care Department, Lille University Medical Center, Avenue Oscar Lambret, F-59037 Lille, France. ^5^ Anesthesiology and Critical Care Department, Dijon University Medical Center, 2 Bd Maréchal de Lattre de Tassigny, F-21000 Dijon, France. SB is the Principal Investigator; SB and PGG conceived the study and led the development of the proposal and the protocol. EL, HD and MOF contributed to study design and to development of the proposal. PB, YEA, RD, MM and OAA drafted the manuscript. All authors read and approved the final manuscript.
Name and contact information for the trial sponsor {5b}	CHU Amiens-Picardie (Amiens University Medical Center). Clinical Research and Innovation Directorate F-80054 Amiens cedex 1 France
Role of sponsor {5c}	CHU Amiens-Picardie: management and analysis of data.

## Introduction

### Background and rationale {6a}

For many years, it was accepted that hemodynamic optimization during major noncardiac surgery was associated with lower rates of perioperative morbidity and mortality. This effect was based on maximization of blood flow (related to cardiac output (CO)), tissue perfusion (related to blood pressure), and thus oxygen delivery (DO_2_) to the tissues [1]. Given that surgery is associated with an increase in oxygen consumption (VO_2_), it can lead to a mismatch between DO_2_ and VO_2_; the body’s metabolism becomes partially anaerobic, with tissue hypoperfusion and then postoperative complications [2]. In this context, the international guidelines now recommend hemodynamic optimization in major noncardiac surgery [3, 4]. This approach is based on the use of fluids, vasopressors, and inotropic drugs. All these medications can trigger adverse reactions, which limits their benefits. Indeed, a few recent studies failed to find a clinical benefit of hemodynamic optimization [5–7]. Similarly, a recent meta-analysis found that hemodynamic optimization did not have medical benefit and was associated with adverse events (such as fluid overload) [8]. There are several possible explanations for these results. Firstly, anesthesia procedures and surgical techniques have improved. Secondly, it has been suggested that hemodynamic optimization does not prevent the onset of anaerobic metabolism [5, 8]. Anaerobic metabolism is thought to be triggered by an increase in VO_2_ and a fall in DO_2_. In such a case, all the determinants of DO_2_ (the hemoglobin level, oxygen saturation, and CO) should be maximized in order to meet the VO_2_ demand. Thirdly, the POMO study demonstrated a beneficial effect of hemodynamic optimization only in patients who attained a VO_2_ value close to that measured preoperatively [5]—suggesting the need for individualized hemodynamic optimization. Fourthly, the postoperative morbidity essentially concerns patients with a low anaerobic threshold (an altered baseline VO_2_), i.e., metabolically fragile patients for whom a DO_2_ maximization strategy could be beneficial [8–10]. Taken as a whole, these results and observations suggest that the risk/benefit balance for hemodynamic management can only be achieved by personalized optimization in identified patients with baseline factors that favor anaerobic metabolism.

There is a large body of literature data on variables that can be used to diagnose and track the DO_2_/VO_2_ balance and changes in tissue perfusion. Most of these variables are derived from blood analyses. The literature data on the various parameters’ value for diagnosing anaerobic metabolism are contradictory [11–15]. Based on the physiological knowledge of VO_2_, DO_2_, and CO_2_ production (VCO_2_), the respiratory quotient is known to be a noninvasive parameter reflecting the balance between DO_2_ and VO_2_ and thus anaerobic metabolism [16]. The respiratory quotient is widely used in physiology and cardiology. In the operating theater, the respiratory quotient can be approximated by measuring the respiratory exchange ratio (RER: the gradient between the inhaled/expired CO_2_ and O_2_ fractions and the minute ventilation). The RER reflects the body’s energy metabolism and anaerobiosis [17]. In a model of hemorrhagic shock, changes in the RER reflect the mismatch between VCO_2_ and VO_2_ and thus anaerobic metabolism. Moreover, treatment of the shock corrected the RER [18]. We recently demonstrated that in major noncardiac surgery, the RER is predictive of hyperlactatemia and the occurrence of postoperative complications [19, 20]. Thus, the RER may identify patients with VO_2_/DO_2_ mismatch and who might benefit from maximization of DO_2_. Given that the inhaled and exhaled fractions of O_2_ and CO_2_ are systematically measured in intubated-ventilated patients in the operating theater, the RER is readily available in all anesthetized patients. It might be of value to additionally consider the RER for individualized hemodynamic management. RER monitoring might limit the occurrence of adverse events associated with excessive hemodynamic optimization (volume overload) by selecting only patients who would be likely to benefit from this optimization strategy.

Thus, we hypothesized that individualized hemodynamic management based on continuous noninvasive measurement of the RER would lower the occurrence of postoperative complications because it provides information on the development of the VO_2_/DO_2_ imbalance and limits excessive hemodynamic treatments.

### Objectives {7}

The study’s primary objective will be to demonstrate that a hemodynamic optimization algorithm based on the RER will lower the incidence of at least one postoperative complication (defined according to the European guidelines) in the 7 days following surgery.

The secondary objectives will be to demonstrate a decrease in the length of hospital stay, the incidence of each complication, the arterial lactate level at the end of the procedure, and mortality at 30 days in patients in the RER group.

### Trial design {8}

**Fig. 1:**
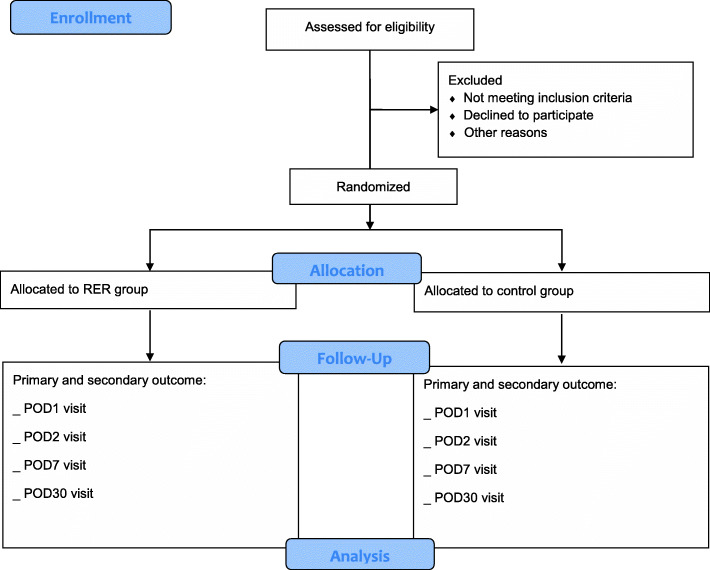
Patient disposition

We are conducting a prospective, open-label, randomized, controlled, comparative, multicenter superiority study of two groups of patients (Fig. 1).

## Methods: participants, interventions, and outcomes

### Study setting {9}

The study will take place in four university medical centers (Amiens University Medical Center, Lille University Medical Center, Caen University Medical Center, Dijon University Medical Center) and a general hospital (Valenciennes General Hospital).

The list of study sites can be obtained from the Clinical Research and Innovation Directorate at Amiens University Medical Center (CHU Amiens- Picardie), F-80054 Amiens cedex 1, France.

### Eligibility criteria {10}

#### Inclusion criteria

1. Abdominal, orthopedic, or vascular surgery with general anesthesia
2. Adult patients
3. American Society of Anesthesiology Physical Status score ≥ II
4. Estimated duration of surgery > 2 h
5. At least two of the following co-morbidities: age > 50, high blood pressure, cardiomyopathy, ECG abnormality, pulmonary edema, smoking, stroke, arteritis, insulin-dependent or noninsulin-dependent diabetes, ascites, chronic kidney failure
6. Written consent
7. Affiliation with a social security scheme

#### Noninclusion criteria

1. Severe untreated arterial hypertension
2. Chronic renal failure on dialysis
3. Acute heart failure
4. Acute coronary syndrome
5. Renal vascular surgery
6. Cardiac surgery
7. Permanent laparoscopy
8. Preoperative acute circulatory failure
9. Refusal to participate
10. Pregnancy
11. Guardianship, curatorship, or incarceration
12. Locoregional anesthesia (spinal and epidural anesthesia)
13. Acute respiratory distress syndrome (PaO_2_/FiO_2_ ratio < 300)
14. Chronic respiratory insufficiency with home oxygen therapy
15. Participation in another clinical study

### The pre-inclusion (enrollment) visit and the associated consenting process {26a}

The pre-inclusion visit must be carried out by an investigator named in the protocol. It will take place during the pre-anesthesia consultation (PAC). All participants who meet the inclusion criteria during the study will be asked to participate in the study. The investigating physician will inform the patient about the study and will answer any questions concerning the study’s objectives, constraints, foreseeable risks, and expected benefits. He/she will also specify the patient’s rights as part of this research and will check the eligibility criteria. Copies of the study information sheet and the consent form are then given to the patient by the investigating physician.

Once the patient has received this information, he/she will be given time to think about whether he/she wishes to participate. The investigating physician is responsible for obtaining the patient’s written, informed consent. The consent form must be signed *before any clinical or paraclinical study procedures* be performed. If the patient agrees to participate, the patient and the investigator write their first and last names clearly and then date and sign the consent form. The various copies of the study information sheet and the consent form are distributed as follows:

A copy of the study information sheet and a copy of the signed consent forms are given to the patient.

The original copies are kept by the investigating physician (even if the patient moves during the course of the research) in a safe place that cannot be accessed by third parties.

At the end of the inclusion period or (at the latest) at the end of the research, a copy of each consent form shall be forwarded to the sponsor or its representative; the investigators will be told in a timely manner how these forms should be forwarded.

### Additional consent provisions for collection and use of participant data and laboratory specimens {26b}

In the context of this research, no additional clinical or paraclinical examinations (i.e., beyond those normally applied for this type of surgery) will be performed.

### Instructions for preparation of requests for an ancillary study

Written informed consent must be provided by each participant in future ancillary studies, if the data collection request is not covered by the informed consent process for the main clinical study. A copy of the institutional review board’s (IRB’s) letter of approval for future ancillary studies will be sent to the OPHIQUE clinical trial’s Data Monitoring Committee. If a separate consent form is required for the ancillary study, a copy of the signed ancillary study consent form for each study participant must be included in the OPHIQUE clinical trial’s records. A data file tracking all signed ancillary consent forms must be maintained by the ancillary study, and an electronic copy of that file must be delivered to the OPHIQUE trial’s Data Monitoring Committee.

### Interventions

#### Explanation for the choice of comparators {6b}

**Fig. 2:**
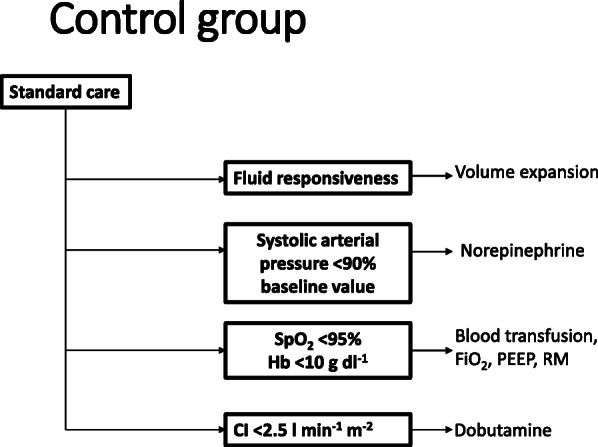
Algorithm for patient management in the control group

In the control group, the patients’ hemodynamic parameters are managed according to international and national guidelines by maintaining blood pressure with norepinephrine, optimizing the stroke volume by fluid challenge, and (if necessary) administering dobutamine [3, 4] (Fig. 2).

Cardiac output is optimized firstly by fluid challenge (maximization) and secondly (in the absence of preload dependency) by intravenous administration of dobutamine to obtain a cardiac index (CI) greater than 2.5 l min^−1^ m^−2^. Dobutamine is administered with an electric syringe pump via a dedicated peripheral venous access. The dose of dobutamine (3 mg per kg body weight) is diluted in the syringe to give a total volume of 50 ml. The initial administration rate is 2.5 ml h^−1^ or 2.5 gamma kg^−1^ min^−1^. If there is no change in the CI, the administration rate is increased in increments of 2.5 ml h^−1^ up to a maximum of 10 ml h^−1^ or 10 gamma kg^−1^ min^−1^. The dobutamine infusion will be decreased if the heart rate rises above 100 bpm or increases by more than 40% of the baseline value.

The systolic arterial pressure (SAP) is maintained at more than 10% of the reference value by continuous intravenous administration of norepinephrine, if necessary. The mean initial infusion rate will be 0.05 μg/kg/min, and the infusion rate will be titrated to maintain the target SAP. The treatment is administered during the operative period only and begins with the induction of anesthesia. Next, the targets are:

- A hemoglobin level greater than 10 g dl^−1^, using blood transfusion
- A pulse oxygen saturation (SpO_2_) value greater than 95%, using a recruitment maneuver, increased FiO_2_, and titration of the positive end-expiratory pressure (Fig. 2)

#### Intervention description {11a}

The RER is calculated from the continuous measurement of inspired and expired gases on the anesthesia ventilator (expressed in %): the inspired fraction of oxygen (FiO_2_), end-tidal fraction of oxygen (FetO_2_), inspired fraction of CO_2_ (FiCO_2_), end-tidal fraction of CO_2_ (FetCO_2_), expired volume (Ve), and inspired volume (Vi). The value is averaged over a 5-min moving window.

Assuming that Vi = Ve during mechanical ventilation in a closed circuit:

$$ {\mathrm{VCO}}_2\ \left(\mathrm{ml}\ {\min}^{-1}\right)=\mathrm{Ve}\times \left({\mathrm{FetCO}}_2-{\mathrm{FiCO}}_2\right) $$

$$ {\mathrm{VO}}_2\ \left(\mathrm{ml}\ {\min}^{-1}\right)=\mathrm{Ve}\times \left({\mathrm{FiO}}_2-{\mathrm{FetO}}_2\right) $$

Thus:

$$ \mathrm{RER}={\mathrm{VCO}}_{2/}{\mathrm{VO}}_2=\left({\mathrm{FetCO}}_2-{\mathrm{FiCO}}_2\right)/\left({\mathrm{FiO}}_2-{\mathrm{FetO}}_2\right) $$

An RER greater than 1.0 indicates anaerobic metabolism [19], and so DO_2_ must be increased. This increase depends on the hemoglobin level, the arterial oxygen saturation, and the CO (Fig. 2).

Cardiac output is optimized firstly by fluid challenge (maximization) and secondly (in the absence of preload dependence) by intravenous administration with dobutamine to obtain a CI greater than 2.5 l min^−1^ m^−2^. Dobutamine is administered with an electric syringe pump via a dedicated peripheral venous line. The dose of dobutamine (3 mg per kg body weight) is diluted in the syringe to give a total volume of 50 ml. The initial administration rate is 2.5 ml h^−1^ or 2.5 gamma kg^−1^ min^−1^. If there is no change in the CI, the administration rate is increased in increments of 2.5 ml h^−1^ up to a maximum of 10 ml h^−1^ or 10 gamma kg^−1^ min^−1^. The dobutamine infusion will be decreased if the heart rate rises above 100 bpm or increases by more than 40% of the baseline value.

The SAP is maintained at more than 10% of the reference value by continuous intravenous administration of norepinephrine, if necessary. The mean initial infusion rate will be 0.05 μg/kg/min, and the infusion rate will be titrated to maintain the target SAP. The treatment is administered during the operative period only and begins with the induction of anesthesia.

Other objectives are:

- A hemoglobin level greater than 10 g dl^−1^, using blood transfusion
- A SpO_2_ greater than 95%, using a recruitment maneuver, increased FiO_2_, and titration of the positive end-expiratory pressure

**Fig. 3:**
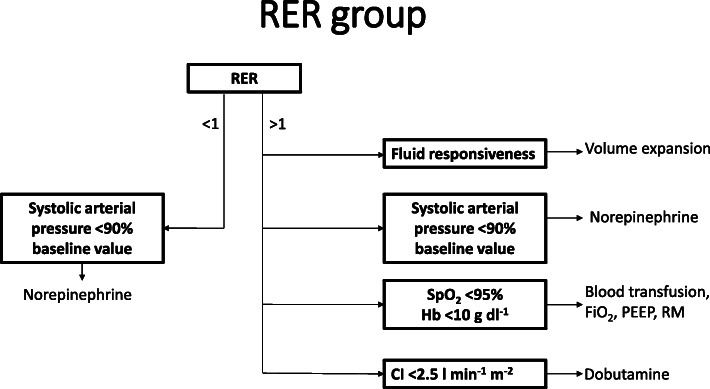
Algorithm for patient management in the interventional group

If RER is lower than 1.0, SAP must be maintained at more than 10% of the reference value by the intravenous continuous administration of norepinephrine (Fig. 3).

To both avoid extreme clinical practices and minimize interference with the trial intervention, study investigators will be strongly encouraged to apply standard measures, as follows:

- Induction of general anesthesia with the use of propofol 2–3 mg kg^−1^, sufentanil 0.2 μg kg^−1^, and cisatracurium 0.15 mg kg^−1^. Inhaled anesthetics or target-controlled infusion of propofol will be used to maintain general anesthesia with a target bispectral index of between 40 and 60, along with intravenous perfusion of sufentanil at 0.1 to 0.2 μg kg^−1^ per hour
- Mechanical ventilation, with a tidal volume of 6–8 ml kg^−1^ predicted body weight, a positive end-expiratory pressure between 5 and 10 cmH_2_O, a FiO_2_ that maintains oxygen saturation ≥ 95%, and the respiratory rate adjusted to maintain an end-tidal CO_2_ between 30 and 35 mmHg
- The core body temperature is maintained at 36.5 °C
- Peroperative use of epidural analgesia is authorized
- Postoperative epidural analgesia is authorized

#### Criteria for discontinuing or modifying allocated interventions {11b}

The study may be discontinued for an individual patient if the latter (or the person designated by the patient) so desires or if decided by the investigator. Every effort will be made to comply with the study protocol. However, the clinician in charge of the patient may deviate from these instructions at any time if he/she considers it necessary. He/she must note this decision and the reason for it in the case report form. If the study is discontinued, no provision is made for patient replacement. Likewise, patients having discontinued the study for whatever reason will receive standard care for the surgery and disease in question.

#### Strategies to improve adherence to interventions {11c}

The coordinating investigator will ensure good adherence to the intervention via the following measures:

- Visits to study centers in order to train study staff in how to monitor the RER in the operating theater (readings, measurements, analysis, and the pathological threshold)
- Visits to study centers to explain the collection of primary and secondary outcomes and the follow-up visits (on postoperative day (POD)1, POD2, POD7, and POD30)
- The hemodynamic optimization protocol is identical to that used in routine care in the operating theater
- Study centers will be regularly contacted by phone in order to ensure good compliance with the research protocol and to answer any questions

Throughout the hospital stay, it is the study team’s responsibility to ensure the proper, timely assessment of outcome measures (including timely visits by the investigator).

#### Relevant concomitant care permitted or prohibited during the trial {11d}

The clinician in charge of the patient’s care may deviate from these instructions at any time if he/she considers it necessary. He/she must note this decision and the reason for it in the case report form. Both treatment groups have access to standard care.

#### Provisions for post-trial care {30}

If the study is discontinued, no provision is made for patient replacement. Likewise, patients having discontinued the study for whatever reason will receive standard care for the surgery and disease in question.

This research is covered by an insurance policy issued by the *Société Hospitalière d’Assurance Mutuelles* insurance company (18 rue Edouard Rochet, F-69372 Lyon cedex 08, France; policy reference 147 731 RC RECH). In accordance with the provisions of Article L1121-10 of the French Public Health Code, the policy covers for nonnegligent harm associated with the study. The policy includes cover for additional health care, compensation, or damages whether awarded voluntarily by the sponsor or through claims pursued in the courts. Incidents judged to arise from negligence (including those due to major protocol violations) are not covered by the study’s insurance policy.

#### Outcomes {12}

The primary study outcome is the proportion of patients in the RER group (vs. the control group) with at least one complication within the 7 days following surgery. Complications were defined according to the guidelines issued by the European Society of Anaesthesia and the European Society for Intensive Care Medicine [21].

##### Neurological complications

- Stroke, documented on CT
- Mental confusion and disorientation in space and time, according to the Confusion Assessment Method for the ICU

##### Respiratory complications

- Acute respiratory distress syndrome (defined as polypnea > 25/min, involvement of the accessory respiratory muscles, or a pH < 7.25), acute lung injury (defined as respiratory distress with a PaO_2_/FiO_2_ ratio < 300 or a PaO_2_ < 80 mmHg under a high-concentration mask), or adult respiratory distress syndrome (defined as respiratory distress with a PaO_2_/FiO_2_ ratio < 200 mmHg)
- Pulmonary embolism, defined as the presence of one or more emboli in the arterial vessels of the nonoperated lung when CT angioscopy is performed
- Prolonged (> 24 h) orotracheal intubation
- Repeated orotracheal intubation

##### Cardiovascular complications

- Acute systolic heart failure confirmed by echocardiographic impairment of the left ventricular ejection fraction vs. the preoperative baseline
- Acute right heart failure confirmed by echocardiographic impairment of right systolic and/or diastolic function
- Acute pulmonary edema with fluid overload confirmed by a clinical examination and echocardiography and that leads to medical treatment
- Acute circulatory insufficiency, defined as treatment with catecholamines (epinephrine, dobutamine, phosphodiesterase inhibitor, levosimendan, and norepinephrine)
- Postoperative myocardial damage: troponin I or T elevation above the 99th percentile
- Myocardial infarction, defined as an elevation in cardiac enzymes (creatine phosphokinase myocardial band, troponin T, or troponin I) plus the appearance of a new Q-wave, ST-segment elevation, or ECG repolarization disorder
- Supraventricular rhythm disorders, defined as the occurrence of atrial flutter or complete arrhythmia by atrial fibrillation and confirmed by ECG
- Ventricular rhythm disorders, defined as the occurrence of ECG-confirmed ventricular tachycardia

Acute renal failure, defined as a postoperative increase of at least 50% and/or 26.5 μmol/l in the creatinine level vs. the preoperative baseline and/or diuresis of less than 0.5 ml kg^−1^ h^−1^ over 6 h (the KDIGO International Society of Nephrology definition) [22]

##### Digestive complications

- Mesenteric ischemia/ischemic colitis documented by CT and/or colonoscopic and/or surgical imaging
- Digestive tract hemorrhage (upper or lower), defined as the occurrence of hematemesis, melena, or rectorrhagia
- Postoperative ileus, defined as a transitory (> 48 h) halt or slowing of intestinal transit

##### Hemorrhagic complications

- Postoperative bleeding
- Disseminated intravascular coagulation
- Transfusion with blood derivatives: red blood cells, platelets, and fresh plasma
- Inflammatory systemic response syndrome, defined as the presence of at least two of the following criteria: (i) body temperature < 36 °C or > 38 °C, (ii) heart rate > 90 beats/min, (iii) respiratory rate > 20 cycles/min or a PaCO_2_ < 32 mmHg in a blood gas analysis, and (iv) leukocyte count < 4000/mm^3^ or > 12,000/mm^3^

##### Infectious complications

- Surgical site infection, defined as the occurrence of wall infection (abscess or purulent discharge) or a documented bacterial infection
- Symptomatic or asymptomatic postoperative urinary tract infection, defined as the presence of a germ (> 10^5^ CFU/mm^3^) in the urine
- Pneumopathy, defined as new lung infiltrates plus at least two of the following: (i) fever > 38.5 °C or hypothermia < 35.5 °C and (ii) leukopenia < 4000 WBCs/mm^3^ or hyperleukocytosis > 12,000 WBCs/mm^3^
- Purulent secretions. Bacteriological confirmation will be made by the presence of microorganisms in bronchial samples (sputum culture > 10^7^ CFU/mm^3^, bronchial aspirate > 10^5^ CFU/mm^3^, bronchoalveolar lavage > 10^4^ CFU/mm^3^, distal protected aspirate or protected specimen brush > 10^3^ CFU/mm^3^)
- Bacteremia, defined as the presence of a positive blood culture for a pathogenic germ
- Infection of a catheter (central venous or arterial), defined as a positive culture of the same microorganism on the catheter (> 10^3^ CFU ml^−1^) and in at least one peripheral blood sample
- Sepsis, defined as life-threatening organ dysfunction caused by a dysregulated host response to infection. For clinical application, organ dysfunction can be represented as an increase in the Sequential [Sepsis-related] Organ Failure Assessment (SOFA) score of 2 points or more, which is associated with an in-hospital mortality rate above 10%
- Septic shock, defined as a subset of sepsis in which particularly profound circulatory, cellular, and metabolic abnormalities are associated with a greater risk of mortality than for sepsis alone. Patients with septic shock can be clinically identified by the requirement for vasopressor to maintain a mean arterial pressure (MAP) of > 65 mmHg and a serum lactate level greater than 2 mmol/l (> 18 mg/dl) in the absence of hypovolemia [23]

*Death* during hospitalization, on POD30, and at 1 year. Each patient’s vital status will be documented via a telephone call 30 days after surgery.

The secondary outcomes are:

- The mean length of stay in each group: the postoperative length of stay in the intensive care unit (ICU) (days) and the overall length of hospital stay (the number of days spent in the hospital until discharge)
- The proportion of patients with complications within the 7 days following surgery in each group
- The mortality rate on POD30 in each group
- The mean total intraoperative IV fluids administered (crystalloids and colloids) in each group
- The mean laboratory criteria (plasma creatinine, lactate, C-reactive protein (CRP), troponin Tc, and brain natriuretic peptide (BNP) and the SOFA score in each group, measured on POD1, POD2, and POD7)

### Participant timeline {13}

The inclusion period is expected to last for 48 months. Several visits are provided for in the protocol. The visits take place when the patient is hospitalized, on the day of inclusion/surgery (POD0), POD1, POD2, POD7, and POD30 (Fig. 1). The visits on POD0, POD1, POD2, and POD7 must be performed on the scheduled day. For the visit on POD30, a margin of 5 days before D30 and 5 days after D30 will be tolerated.

#### Allocation

The patients will be randomized by an investigator named in the protocol. After checking the patient’s inclusion criteria and consent, the investigator connects to the secure (SSL) website https://recherche-clinique.chu-amiens.fr/CSOnline/ and creates a patient file in the database (assignment of the patient code). The investigator will have to enter the requested data in order to check the inclusion and noninclusion criteria. If all the requested data are entered and are consistent, the investigator will be given the result of the randomization as either “standard management” or “RER.” If any of the data are erroneous or data, an error message will indicate which corrections must be made so that the patient can be randomized.

#### Follow-up visits

Five follow-up visits are planned: on the day of inclusion/surgery (POD0), POD1, POD2, POD7, and POD30. The visits on POD1, POD2, POD7, and POD30 will be performed by an anesthesiologist who is blinded to the group allocation and has not managed the patient in the operating theater. These visits enable the progressive, consecutive collection of the study data.

Participation in the study will not change how patients are managed during their stay in the ICU and in hospital generally, other than compliance with the hemodynamic optimization protocol. As part of their care, patients always provide several blood samples—including at least one per day during the first week.

#### End-of-study visit

The end-of-study visit takes place on POD30. The purpose of this visit is to collect study data generated since the previous visit (POD7). The variables documented will be the same as those documented at the POD7 visit. The end of the study is defined as the last study visit by the last person participating in the study. No particular post-study treatments are planned for study participants.

### Study timeline for participants

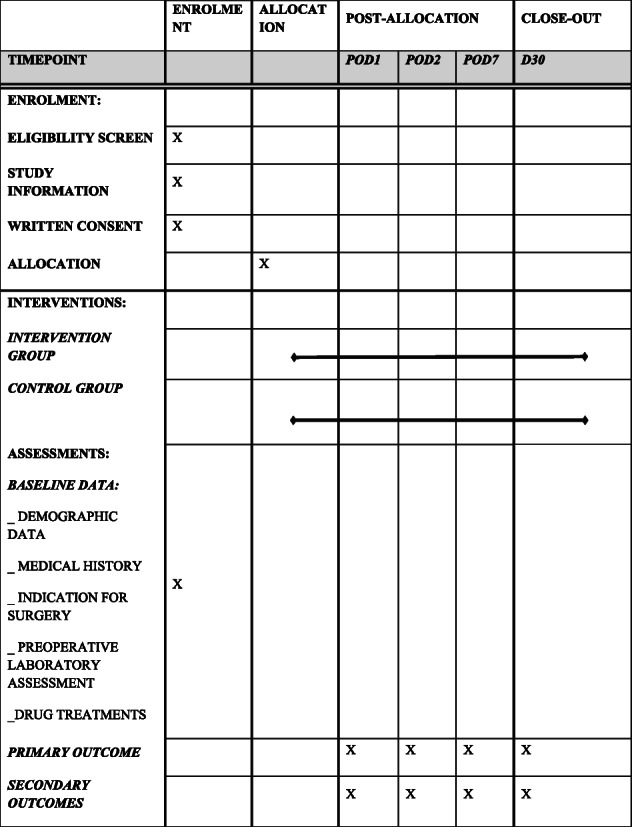



### Sample size {14}

According to the literature data, the postoperative complication rate in the target population is between 30 and 50% [24]. The inclusion of 170 patients in each arm would show that RER-based hemodynamic optimization reduces these complications in a clinically significant manner (by a third; from 50% in the control group to 35% in the interventional arm). These calculations were performed with an alpha risk of 5%, a power of 80%, and a complication rate of 50% in the control arm.

Furthermore, an interim analysis is planned halfway through the inclusion process. Hence, the final study population should be 344 patients. Based on these results and after taking account of potential loss to follow-up, we plan to include 350 patients (175 per arm). If 175 patients are included in each arm, the threshold for statistical significance in a one-sided test will be set to *p* < 0.003 (according to the Lan and Demets spending function, using the seqdesign SAS procedure). Premature study termination for efficacy could thus be considered.

### Recruitment {15}

The feasibility of this project is guaranteed by (i) the relatively large number of potentially eligible patients treated in the various centers and (ii) the involvement of a multicenter group whose members have been working together on hemodynamic optimization research projects for many years.

Number of eligible patients per center:

Amiens University Medical Center: 2 to 3 inclusions per month

Lille University Medical Center: 1 inclusion per month

Caen University Medical Center: 1 inclusion per month

Dijon University Medical Center: 2 to 3 inclusions per month

Valenciennes General Hospital: 1 inclusion per month

### Assignment of interventions: allocation

#### Sequence generation {16a}

Patients will be randomized into the two parallel, open-label groups using Clinsight® software, as implemented by a data manager from the Clinical Research and Innovation Directorate at Amiens University Medical Center. Randomization by minimization will be performed.

The randomization will be stratified:

- By center
- By the type of surgery (orthopedic, vascular, or abdominal)

Patients will be randomly assigned to one of the study groups via centralized randomization by minimization, in order to check for stratification factors and balance the size of the groups. This will also make it possible to check patient eligibility and send randomization information to the investigator for each patient.

After entering their username and password, the investigators will connect to the study randomization site on the day of inclusion and check compliance with the inclusion and noninclusion criteria. If no inconsistencies are found, the result of the randomization will be displayed as “standard management” or “RER.”

#### Concealment mechanism {16b}

Prior to the provision of informed consent, participants (and, if applicable, their legal guardians) will be given full information about the study by the researchers at each study site. The provision of study information will be recorded in the Clinsight® software by the research assistants. After the baseline survey, eligible participants will be randomized 1:1 into two groups.

#### Implementation {16c}

The pre-inclusion visit is carried out by an investigator named in the protocol. It will take place during the PAC. All selected patients meeting all the inclusion criteria and none of the noninclusion criteria will be invited to participate in the study.

### Assignment of interventions: blinding

#### Who will be blinded {17a}

Although the operating anesthetists cannot be blinded to group assignments, much attention will be given to ensuring strict blinding during the postoperative, data collection, and data analysis periods. The surgeons and ICU physicians and nurses are blinded to group allocation. Only the anesthetist in charge of the patient during the surgery will be aware of the group allocation.

#### Procedure for unblinding if needed {17b}

An unblinding procedure is not necessary and not planned.

### Data collection and management

#### Plans for assessment and collection of outcomes {18a}

All data will be continuously recorded on an electronic case report form (eCRF) by a clinical data manager who is blinded to the group allocation. The eCRF was created by study investigators. All the information required by the protocol must be recorded as and when it is obtained in the eCRF, and any missing data must be explained:

- “NK” for “not known”
- “ND” for “not determined”
- “NA” for “not applicable”

Any access to the study data and any changes will be tracked by the Clinsight® software (Ennov Clinical).

1. Preoperative data
  - Demographic data
  - Medical history (high blood pressure, diabetes, kidney failure, etc.), and the indication for surgery (neoplastic disease or not)
  - Preoperative laboratory assessment (including plasma creatinine and CRP levels)
  - Drug treatments (antihypertensive drugs, antidiabetics, diuretics, etc.)
2. Intraoperative data
  - *Anesthetic data:*

I Hypnotics, opioids, and muscle relaxants used
II Volume of fluids (crystalloids and colloids) used
III Number of labile blood products used
  (b) *Hemodynamic data (every 30 min):*
I SAP, DAP, and MAP
II Heart rate
III Bispectral index
IV Temperature
V Cardiac output
VI Pulse pressure variation
VII Total amount of ephedrine and norepinephrine used
VIII Total amount of dobutamine used
IX Intraoperative diuresis
  (c) *Surgical data:*
I Surgical indication and type of surgery (abdominal, vascular, or orthopedic)
II Duration of surgery
III Volume of blood loss
IV Intraoperative surgical complications

3. Postoperative data

- Length of stay in the recovery room
- Patient discharge (to a surgical department, the ICU, or the recovery room)
- Laboratory assessment:

I Kidney function: serum creatinine and urea levels on POD1, POD2 and POD7, and renal replacement therapy
II Blood lactate and plasma creatinine, CRP, BNP, and troponin Tc on POD1, POD2, and POD7

(d) Standard clinical data (heart rate, blood pressure, peripheral O_2_ saturation, and respiratory rate)
(e) Diuresis on POD1, POD2, and POD7
(f) KDIGO scores on POD1, POD2, and POD7
(g) Postoperative complications
(h) Length of stay in the ICU and in hospital
(i) 30-day postoperative mortality

#### Plans to promote participant retention and complete follow-up {18b}

At each visit, participants will be reminded of the importance of their participation in the study and the need to be continuously contactable and available for monitoring until the end of the follow-up period. We will make sure that appointments for study visits are available at different times of the day and on different days of the week. Saturday visits will also be available for participants who cannot attend during the week.

Participants who withdraw from the trial will be contacted (with their prior permission) by phone on POD30 in order to check on whether they have developed postoperative complications.

#### Data management {19}

All data will be continuously recorded on the eCRF by a clinical data manager who is blinded to patient allocation. Data collected directly during the patient’s follow-up will be entered (single-entry) into an electronic case report form by the principal investigator (or by authorized persons referenced on the task delegation list) on the https://recherche-clinique.chu-amiens.fr/CSOnline/ website.

The pre-configured consistency tests will check the quality of the data. If inconsistencies are detected, queries will be sent to the investigator.

The data coding will be given on a separate sheet in the electronic case report form. Data will be entered initially as the actual numerical value or the actual categorical variable.

At the end of the study, the Clinsight® database (Ennov Clinical) will be frozen by a data manager at Amiens University Medical Center’s Clinical Research and Innovation Directorate. The centralized data will then be analyzed using SAS® statistical software (version 9.4, SAS Institute Inc., Cary, NC, USA).

#### Confidentiality {27}

In accordance with the legislative provisions in force (articles L.1121-3 and R.5121-13 of the French Public Health Code), people with direct access to source data will take all the necessary precautions to ensure the confidentiality of information relating to the study drugs, research, participants (especially the latter’s names) and results. Like the investigators, these people are subject to a professional duty of confidentiality.

During or after the study, the data collected on the participants will be sent to the sponsor by the investigators (or any other specialist staff) and pseudonymized. The data must in no case show the study participants’ names or addresses.

Data confidentiality will be ensured by coding the participants’ information as follows:

- Each participant’s initials: the first letter of the family name and the first letter of the first name

- A 5-digit code number: the first two digits correspond to the center number and the final three digits correspond to the incremental inclusion number (from 001 upwards)

The sponsor will ensure that each study participant has given his/her written consent to access to his/her personal data required for the study’s quality control procedures.

### Plans for collection, laboratory evaluation, and storage of biological specimens for genetic or molecular analysis in this trial/future use {33}

Blood samples will be collected via venipuncture and arterial lines and then analyzed in our clinical biochemistry laboratory. We shall assay serum creatinine, lactatemia, CRP, BNP, and troponin Ic on POD1, POD2, and POD7. The samples will be discarded shortly afterwards. No biological specimens will be stored for future studies. No genetic or molecular analyses of human DNA are planned.

### Statistical methods

#### Statistical methods for primary and secondary outcomes {20a}

The study’s primary objective is to demonstrate that the incidence of postoperative complications is lower in an interventional group (patients monitored using a hemodynamic algorithm that incorporates the RER) than in a control group (patients treated according to standard practice). To summarize the characteristics of the study population, quantitative variables will be described as the mean ± standard deviation and/or the median [interquartile range]. Qualitative variables will be described as the frequency [95% confidence interval].

##### Analysis of the primary outcome

The null hypothesis will be rejected in favor of the alternative hypothesis (i.e., there is a difference) using a chi-squared test or Fisher’s test, depending on the frequency of complications (*n* < 5 in a cell of the contingency table), with a two-sided alpha risk of 5%.

##### Analysis of the secondary outcome

The two arms will be compared with regard to the total volume of intraoperative fluids and the length of stay, using Student’s test or the Mann-Whitney test for normally distributed data.

The two arms will be compared with regard to the changes over time in the SOFA score and the serum NT-pro-BNP, troponin TC, CRP, lactate, and creatinine levels, using a mixed-model analysis of variance. Post hoc comparisons will be performed after Hochberg adjustment. If the data are not normally distributed, they will be log-transformed or otherwise transformed.

The intergroup difference in the incidence of each complication (including mortality) will be compared in a chi-squared test or Fisher’s test.

All statistical analyses will be performed using SAS® software (version 9.4). The threshold for statistical significance will be set to *p* < 0.05 for both the primary and secondary outcomes. The statistical criteria for premature termination of the study are listed above in the statistical analysis section (*p* < 0.003).

#### Interim analyses {21b}

An interim analysis is planned half way through the inclusion process (*n* = 175 out of 350).

#### Methods for additional analyses (e.g., subgroup analyses) {20b}

No additional analyses are planned.

#### Methods in analysis to handle protocol nonadherence and any statistical methods to handle missing data {20c}

For the primary outcome, we expect the proportion of missing data to be below 5%. We will not perform multiple imputations for the primary outcome unless more than 5% of the data are missing.

The secondary outcomes will be assessed in the full analysis set.

#### Plans to give access to the full protocol, participant-level data, and statistical code {31c}

The full protocol is available from ClinicalTrials.gov (Identifier: NCT03852147. February 25, 2019).

Participant-level data and statistical code are available on request from the Clinical Research and Innovation Directorate at Amiens University Medical Center.

### Oversight and monitoring

#### Composition of the coordinating center and trial steering committee {5d}

**Principal investigator and research physician**

Dr Stéphane BAR, M.D

Anesthesiology and Critical Care Department, Amiens University Medical Center, Rond-point du Professeur Christian Cabrol, F-80000 Amiens, France.

*Roles and responsibilities:*

Design and conduct of the OPHIQUE study

Preparation of the protocol and any revisions

Preparation of the investigator’s brochure and case report forms

Organization of steering committee meetings

Management of the clinical trials office

Publication of study reports

**Steering committee**

(see the title page for members)

*Roles and responsibilities:*

Agreement of the final protocol

All lead investigators will be steering committee members

Patient recruitment and liaison with the principle investigator

Review of the study’s progress and (if necessary) agreement of amendments to the protocol and/or investigator’s brochure to facilitate the smooth running of the study

**Sponsor**

CHU Amiens-Picardie

Clinical Research and Innovation Directorate

F-80054 Amiens cedex 1

France

Tel.: +33-322-088-371; Fax: +33-322-089-645

**Pauline Morin, data manager**

Clinical Research and Innovation Directorate

F-80054 Amiens cedex 1

France

*Roles and responsibilities:*

Maintenance of the trial’s IT system

Data entry and verification

**Clinical Research Vigilance Unit:**

Professor Jean-Marc Chillon

CHU Amiens-Picardie

F-80054 Amiens cedex 1

France

Tel: +33-322-088-371 (ext. 88390); Fax: +33-322-089-645

**Lead investigators**

In each participating center, a nominated lead investigator (a senior anesthesiologist) will be responsible for patient identification, patient recruitment, data collection, completion of CRFs, patient follow-up, and compliance with the study protocol and the investigator’s brochure.

#### Composition of the data monitoring committee, its role and reporting structure {21a}

Data Monitoring Committee

Momar Diouf, biostatistician

Pauline Morin, data manager

Salah Mattoug, project manager

Clinical Research and Innovation Directorate

CHU Amiens-Picardie

F-80054 Amiens cedex 1

France

Tel: +33-322-088-371 Fax: +33-322-089-645

*Roles and responsibilities:*

Study planning

Organization of steering committee meetings

Training session: each center’s study personnel will be trained centrally in the study requirements, standardized measurement of the RER, requirements for outcome collection, advice on adherence, and eliciting information from study participants in a uniform, reproducible manner. The training session will also cover data entry, responses to data discrepancy queries, and general information about obtaining high-quality research data.

Provision of an annual risk report to the French Agency for Health Product Safety (ANSM) and the IRB

Reporting to the ANSM on any serious unexpected suspected adverse events.

Management of the trial master file

Budget administration and contractual issues with individual centers

Advice for lead investigators

Audit of feedback forms and decisions on when site visits should occur

Assistance with IRB applications

Data verification

Randomization

#### Adverse event reporting and harms {22}

The investigator assesses the seriousness of each adverse event and will notify the sponsor without delay from the day on which he/she becomes aware of any serious adverse event or new development, if it occurs:

- After the date on which the consent form is signed
- During the patient’s follow-up

All such events must be monitored until they have completely resolved. Additional information (on a supplementary reporting form) concerning the progression of the event (if not mentioned in the first report) will be sent to the sponsor by the investigator.

The sponsor or the pharmacovigilance unit will report developments in the study in a timely manner to:

- The French Agency for Health Product Safety (ANSM)
- The competent IRB. If necessary, the IRB will check that the study participants have been informed of the adverse effects and have not withdrawn their consent:

1. For an unexpected serious adverse reaction resulting in death or endangerment of life, without delay from the day on which the sponsor becomes aware of it
2. For other unexpected serious adverse effects, no later than 2 weeks from the day on which the sponsor becomes aware of them

The sponsor shall send the ANSM a follow-up report with relevant additional information on unexpected serious adverse reactions. In the case of a suspected unexpected serious adverse reaction resulting in death or life-threatening injury, this additional information shall be notified within a week of the report mentioned in (1). In the other cases of a suspected serious unexpected adverse effect and if new information (as defined in Article L.1123-10) has emerged, the relevant additional information must be sent within a week of the deadline mentioned in (2).

If necessary, the IRB will check that the study participants have been informed of the adverse effects and have not withdrawn their consent. The sponsor and the investigator shall take appropriate urgent action. The sponsor shall inform the competent authority and the IRB.

On the anniversary date of the first inclusion, the sponsor shall draw up a safety report, including (i) a list of serious adverse events that might be related to the research (including expected and unexpected serious events) and (ii) a concise, critical analysis of the study participants’ safety.

This report shall be sent to the ANSM and the IRB within 60 days of the anniversary date of the first inclusion.

This information will also be submitted to the data monitoring and safety committee.

All adverse events and adverse drug reactions will be reported in publications of the trial’s data. The proportion of patients in each group experiencing at least one adverse event will be reported.

Data on harms will be collected in a nonsystematic manner throughout the patient’s follow-up period. The investigator must notify the sponsor without delay from the day on which he/she becomes aware of any harms. Harms potentially related to the drugs and procedures used are described below:

The following terminologies have been used to classify the frequency of side effects: very common (≥ 1/10), common (≥ 1/100 but < 1/10), uncommon (≥ 1/1000 but < 1/100), rare (≥ 1/10,000 but < 1/1000), very rare (< 1/10000), and not known (frequency cannot be estimated from the data available).

The harms that may occur with the use of dobutamine are as follows:

*Blood and lymphatic system disorders*

Common: eosinophilia, inhibition of thrombocyte aggregation (only with infusion over many days)

*Metabolism and nutrition disorders*

Rare: hypokalemia

*Nervous system disorders*

Common: headache

*Cardiac disorders/vascular disorders*

Common: increase in heart rate of > 30 beats/min

Common: blood pressure increased by > 50 mmHg

Patients with high blood pressure are more likely to experience a higher voltage increase

Decreased blood pressure, ventricular dysrhythmia, dose-dependent ventricular extrasystoles Increased ventricular rate in patients with atrial fibrillation.

Vasoconstriction in some patients previously treated with beta blockers

Anginal pain, palpitations

Most patients have an increase in systolic pressure (10 to 20 mmHg)

Uncommon: ventricular tachycardia, ventricular fibrillation

Rare: bradycardia, myocardial ischemia, myocardial infarction, cardiac arrest

Not known: decreased pulmonary capillary pressure

Dose-dependent side effects rarely occur with a dosage below 10 μg/kg/min; a dose of 40 μg/kg/min has been administered in some cases, without significant adverse effects

Hypertensive/hypotensive decompensation, intracavity pressure gradient, palpitations

Not known: stress cardiomyopathy

*Respiratory, thoracic, and mediastinal disorders*

Common: bronchospasm, shortness of breath

*Gastrointestinal disorders*

Common: nausea

*Skin and subcutaneous tissue disorders*

Common: exanthema

Rare: petechial bleeding

*Musculoskeletal and connective tissue disorders*

Common: chest pain

*Kidney and urinary tract disorders*

Common: increased urgency at high infusion doses

*General disorders and administration site abnormalities*

Common: fever, phlebitis at the injection site

In the event of accidental paravenous infiltration, local inflammation may develop

Very rare: skin necrosis

*Other events*

Nervousness, nausea, headache, paresthesia, tremors, urinary urgency, feeling hot and anxiety, myoclonic spasm

The harms that may occur with the use of norepinephrine are as follows:

Common: anxiety

Common: breathlessness

Common: headache

Common: tremor

Rare: skin necrosis

Rare: perivenous extravasation.

Rare: retrosternal pain

Rare: pharyngeal pain

Rare: photophobia

Rare: paleness

Rare: excessive sweating

Rare: vomiting

Rare: tachycardia

Rare: bradycardia

The harms that may occur with the use of ephedrine are as follows:

Common: palpitation

Common: arterial hypertension

Rare: coagulation disorder

Rare: nervousness

Rare: tremor

Rare: anxiety

Rare: insomnia

Rare: mental confusion

Rare: irritability

Rare: depression

Rare: urinary retention

Rare: hypersensitivity

Rare: acute glaucoma attack

All adverse events and drug-related will be reported in trial publication. The proportion of patients experiencing any adverse event in each group will be reported.

#### Frequency and plans for auditing trial conduct {23}

A clinical research associate mandated by the sponsor will visit each center at the time the study is set up, one or more times during the study (depending on the inclusion rate), and at the end of the research. During these visits, the following elements will be reviewed: informed consent, compliance with the research protocol and the procedures defined therein, quality of the data collected in the electronic case report form, accuracy, missing data, consistency of data with source documents (medical records, appointment books, original laboratory results, etc.), and management of any products.

A written monitoring report will be required after each visit.

#### Plans for communicating important protocol amendments to relevant parties (e.g., trial participants, ethical committees) {25}

Any protocol amendments must be authorized by the IRB prior to implementation. The IRB will be informed of nonsubstantial amendments (i.e., those that do not have a significant impact on any aspect of the research).

Prior to submission to the IRB, all amendments are to be validated by the sponsor and by all research stakeholders affected by the amendment. All amendments to the protocol must be made known to all investigators participating in the study. The investigators undertake to comply with the protocol. Any amendment that modifies patient management or the study’s benefits, risks, and constraints requires a new study information sheet and a new consent form, which must be processed according to the above-mentioned procedures. The following study documents will be archived in accordance with good clinical practice.

1. By the investigating physicians:

- For a period of 15 years after the end of the study (for studies related to medicines, medical devices or in vitro diagnostics, or for studies not related to a product mentioned in Article L.5311-1 of the French Public Health Code):

I The protocol and any amendments
II Case report forms (copies)
III Source files for study participants having given their written consent
IV All the other documents and correspondence related to the research

(b) For a period of 30 years after the end of the study

I The participants’ original signed informed consent forms

All these documents are placed under the responsibility of the investigator for the prescribed period of archiving.

2. By the sponsor

- For a period of 15 years after the end of the study (for studies related to medicines, medical devices or in vitro diagnostics, or for studies not related to a product mentioned in Article L.5311-1 of the French Public Health Code)
- For a period of 30 years after the end of the study

I Copies of the participants’ original signed informed consent forms
II Documents concerning serious adverse events

All such documents are the sponsor’s responsibility for the legally stipulated period of archiving.

No documents are to be removed or destroyed without the sponsor’s agreement. At the end of the legally stipulated period of archiving, the sponsor will be consulted prior to destruction of the documents. All data, documents, and reports may be subject to audit or inspection.

#### Dissemination plans {31a}

The data provided by the investigative centers will be analyzed by a biostatistician from the Clinical Research and Innovation Directorate at Amiens University Medical Center. This analysis will give rise to a written report submitted to the sponsor, who will forward it to the IRB and the competent authority.

Any written or oral communication of the results of the research must be first be approved by the principal investigator and, where appropriate, any committee set up for the purposes of the study.

The publication of the principal results will mention the name of the sponsor, all investigators who included or followed up patients in the research, and the methodologists, biostatisticians, and data managers who participated in the research. The international rules for writing and publication will be taken into account (The Uniform Requirements for Manuscripts of the ICMJE, April 2010). In accordance with the French Parliamentary Act 2002-303 dated March 4, 2002, the study participants will be informed (on request) of the study’s overall results.

## Discussion

In the operating theater, the RER is potentially a continuously available, easy-to-read, indirect marker of tissue hypoperfusion and postoperative complications. In contrast to conventional tissue perfusion variables, the RER does not require repeated, invasive measurements of venous and arterial blood gas levels; indeed, the repetition of blood gas measurements may decrease reproducibility. Since the RER can diagnose patients with DO_2_/VO_2_ mismatch, it enables personalized hemodynamic management and thus may limit the side effects of excessive hemodynamic optimization (volume overload, vasoconstriction, etc.). Thus, the RER might help to tailor hemodynamic optimization to each patient as a function of the type of surgery and the metabolic requirement. If this approach works, the RER could be used routinely in the operating theater to improve the management of patients undergoing major surgery.

One limitation of the present study is that the operating anesthetists are not blinded to the group assignments. Hence, we have paid as much attention as possible to strict blinding during the postoperative, data collection and data analysis periods. The surgeons, ICU physicians, and nurses are blinded to the group allocation. Only the anesthetist in charge of the patient during the surgery knows the group allocation. Moreover, this is the first large, randomized, controlled trial of the effectiveness of the RER in the operating theater.

## Trial status

This is protocol version 1.6, dated March 3, 2020. Recruitment started on December 26, 2018. To date, 120 participants have been randomized. The estimated date for the completion of recruitment is January 2023.

## Supplementary Information

**Additional file 1.** WHO Trial Registration Data Set.txt.

**Additional file 2.** Copy of the informed consent form.txt.

## Abbreviations

ANSM: French Agency for Health Product Safety
CI: Cardiac index
CO: Cardiac output
DAP: Diastolic arterial pressure
DO_2_: Arterial oxygen delivery
FetO_2_: End-tidal fraction of oxygen
FetCO_2_: End-tidal fraction of CO_2_
FiO_2_: Inspired fraction of oxygen
FiCO_2_: Inspired fraction of CO_2_
MAP: Mean arterial pressure
PAC: Pre-anesthesia consultation
RER: Respiratory exchange ratio
SAP: Systolic arterial pressure
SpO_2_: Pulse oxygen saturation
SOFA: Sequential [Sepsis-related] Organ Failure Assessment
VCO_2_: CO_2_ production
VO_2_: Oxygen consumption
