# Supplementary material for: Individualized hemodynamic optimization guided by indirect measurement of the respiratory exchange ratio in major surgery: study protocol for a randomized controlled trial (the OPHIQUE study)
Source: Trials. 2020 Nov 23;21:958. doi: 10.1186/s13063-020-04879-x (PMC7682128; doi:10.1186/s13063-020-04879-x)
Supplement: Supplementary file 1 — Additional file 1. WHO Trial Registration Data Set.txt. [file 13063_2020_4879_MOESM1_ESM.docx]

| **Data category** | **Information** |
| --- | --- |
| **Primary registry and trial identifying number** | ClinicalTrials.gov identifier: NCT03852147. |
| **Date of registration in primary registry** | Registered on February 25, 2019. |
| **Secondary identifying numbers** | Programme Hospitalier de Recherche Clinique (PHRC) Inter-régional GIRCI Nord-Ouest 2017 (API17-03).  CPP Ile de France II; reference: ID-RCB/ EUDRACT: 2018-A00593-52 |
| **Source(s) of monetary or material support** | The trial was funded by Programme Hospitalier de Recherche Clinique (PHRC) Inter-régional GIRCI Nord-Ouest 2017 (API17-03). |
| **Primary sponsor** | CHU Amiens-Picardie (Amiens University Medical Center).  Clinical Research and Innovation Directorate |
| **Contact for public queries** | CHU Amiens-Picardie (Amiens University Medical Center).  Clinical Research and Innovation Directorate |
| **Contact for scientific queries** | Stéphane BAR, M.D, Amiens University Medical Center, Amiens, France. |
| **Public title** | Hemodynamic optimization guided by indirect measurement of the respiratory exchange ratio. |
| **Scientific title** | Individualized hemodynamic optimization guided by indirect measurement of the respiratory exchange ratio in major surgery: study protocol for a randomized controlled trial (the OPHIQUE study). |
| **Countries of recruitment** | France |
| **Health condition(s) or problem(s) studied** | Hemodynamic management |
| **Intervention(s)** | **Control group:**  the patients’ hemodynamic parameters are managed according to international and national guidelines by maintaining blood pressure with norepinephrine, optimizing the stroke volume by fluid challenge, and (if necessary) administering dobutamine.  **Interventional group:**  The RER is calculated from the continuous measurement of inspired and expired gases on the anesthesia ventilator (expressed in %):  RER = VCO_2/_VO_2_ = (FetCO_2_-FiCO_2_)/(FiO_2_-FetO_2_)  An RER greater than 1.0 indicates anaerobic metabolism [19], and so DO_2_ must be increased. This increase depends on the hemoglobin level, the arterial oxygen saturation, and the CO. |
| **Key inclusion and exclusion criteria** | Inclusion criteria  1. Abdominal, orthopedic or vascular surgery with general anesthesia  2. Adult patients  3. American Society of Anesthesiology Physical Status score ≥II  4. Estimated duration of surgery >2 hours  5. At least two of the following co-morbidities: age >50, high blood pressure, cardiomyopathy, ECG abnormality, pulmonary edema, smoking, stroke, arteritis, insulin-dependent or non-insulin-dependent diabetes, ascites, chronic kidney failure.  6. Written consent  7. Affiliation with a social security scheme.  Non-inclusion criteria   1. Severe untreated arterial hypertension 2. Chronic renal failure on dialysis. 3. Acute heart failure. 4. Acute coronary syndrome. 5. Vascular surgery with renal plasty. 6. Cardiac surgery. 7. Permanent laparoscopy. 8. Preoperative acute circulatory failure 9. Refusal of patient participation. 10. Pregnant woman. 11. Patient under guardianship or curatorship or private of public law. 12. Anesthesia only with loco-regional technique (spinal and epidural anesthesia). 13. Acute respiratory distress syndrome (PaO_2_/FiO_2_ ratio < 300). 14. Chronic respiratory insufficiency with home oxygen therapy. 15. Patient included in another study. |
| **Study type** | Prospective, open-label, randomized, controlled, comparative, multicenter, superiority study of two groups of patients. |
| **Date of first enrolment** | December 26^th^, 2018 |
| **Target sample size** | 350 |
| **Recruitment status** | Recruiting |
| **Primary outcome(s)** | The proportion of patients in the RER group (vs. the control group) with at least one complication within the 7 days following surgery. |
| **Key secondary outcomes** | - The mean length of stay in each group: the post-operative length of stay in the intensive care unit (ICU) (days) and the overall length of hospital stay (the number of days spent in hospital until discharge). - The proportion of patients with complications within the 7 days following surgery in each group. - The mortality rate on POD30 in each group. - The mean total intraoperative IV fluids administered (crystalloids and colloids) in each group. - The mean laboratory criteria (plasma creatinine, lactate, C-reactive protein (CRP), troponin Tc, and brain natriuretic peptide (BNP) and the SOFA score in each group, measured on POD1, POD2 and POD7. |
