# Supplementary material for: Individualized hemodynamic optimization guided by indirect measurement of the respiratory exchange ratio in major surgery: study protocol for a randomized controlled trial (the OPHIQUE study)
Source: Trials. 2020 Nov 23;21:958. doi: 10.1186/s13063-020-04879-x (PMC7682128; doi:10.1186/s13063-020-04879-x)
Supplement: Supplementary file 2 — Additional file 2. Copy of the informed consent form.txt. [file 13063_2020_4879_MOESM2_ESM.docx]

**STUDY INFORMATION SHEET FOR PARTICIPATION IN A CLINICAL TRIAL**

| **Study title:** | | | **Individualized hemodynamic optimization guided by indirect measurement of respiratory exchange ratio in major surgery: study protocol for a randomized controlled trial (the OPHIQUE study)** | | | |
| --- | --- | --- | --- | --- | --- | --- |
|  |  |  |  | | | |
|  | | | |  |  | |
| **Sponsor**: **CHU Amiens-Picardie** | | | |  | **Coordinating Investigator:** | |
|  |  | F-80054 Amiens cedex 1, France | |  | Dr. Stéphane Bar |  |
|  |  | Tel.: +33-322-088-051 | |  | Anesthesia and Resuscitation Department | |
|  |  | Fax.: +33-322-088-057 | |  | CHU Amiens-Picardie | |

F-80054 Amiens cedex 1, France

Tel.: +33-322-087-906

bar.stephane@chu-amiens.fr

Madam, Sir,

Professor/Dr……………………………………………… has invited you to participate in a clinical study sponsored by CHU Amiens-Picardie [Amiens-Picardie University Medical Center].

Before making your decision, it is important that you read these pages carefully as they will provide you with all the necessary information on the various aspects of the study. Do not hesitate to ask [your physician] if you have any questions.

Your decision to participate in the study is entirely voluntary. **You may withdraw from this study at any time.** If, during the study, new information becomes available that may influence your decision to continue participating, you will be notified immediately.

1. **Why is this research being performed?**

The occurrence of events and complications during or after high-risk surgery is related to the balance between the body’s oxygen supply and its oxygen requirements.

During surgery, two of the anesthesiologist’s major concerns are ensuring the stability of the patient’s cardiac and vascular variables (blood pressure, heart rate, blood oxygen level) and supplying enough oxygen to the cells. At present, it is difficult for anesthesiologists to identify the best guide to these variables during surgery.

The objective of the present study is to analyze the data on the changes in levels of carbon dioxide (CO_2_) and oxygen (O_2_) - the gases you breathe. These levels are often monitored during surgery; they reflect your body's oxygen needs and therefore the ability to supply oxygen to the organs (the heart, kidney, brain, etc.). So, variations in these levels might be associated with the occurrence of events and complications during or after surgery.

1. **What is the study’s objective?**

The study’s main objective is to determine whether there is a difference in post-surgery complications between two groups of patients (**the experimental group and the standard group)**, whose cardiac and vascular functions are optimized in different ways.

In the standard group, the patients will be managed according to the department’s usual protocol. In the experimental group, the patients will be managed according to measurements of variations in the levels of CO_2_ and O_2_ – the gases inhaled and exhaled by the lungs. These levels might inform the physician in real time about the balance between oxygen transport by the blood and oxygen consumption by the cells. This will allow the surgeon to adapt the treatment to suit the patient’s needs during the operation.

1. **How will this research be carried out?**

If you agree to participate in the study, the first step is your assignment to a study group: the experimental group in which the optimization of your body's oxygen transport will be based on measurements of inhaled and exhaled CO_2_ and O_2_ levels, or the standard group that does not involve this analysis. This allocation will be done randomly (by drawing lots). After your operation (during specific follow-up visits), we will record any post-operative complications that you may develop.

Five follow-up visits will be scheduled: on the day of your inclusion (day 0), and on post-inclusion days 1, 2, 7, and 30. The visits on post-inclusion days 1, 2, 7, and 30 will be performed by a different anesthesiologist – not the one who took care of you in the operating room. These visits will allow us to progressively and consecutively record the medical data required for this study.

This study will not lead to any change in your care during your stay in the intensive care unit, apart from compliance with the protocol for optimizing cardiac and vascular function variables. As part of your normal care, several blood samples will be collected (including at least one a day during the first week after surgery).

The end-of-study visit will take place 30 days after your operation.

1. **Who can participate in the study?**

Patients who participate in the study will be adults (aged 18 or over) undergoing abdominal, orthopedic or vascular surgery with general anesthesia, have social security coverage, and meet at least two of the following criteria: age 50 or over, high blood pressure, heart disease, abnormal electrocardiogram, acute pulmonary edema, smoking, stroke/transient ischemic attack, obstruction of the arteries that irrigate the lower limbs, diabetes (whether insulin-dependent or not), ascites (presence of non-bloody fluid in the abdominal cavity), and chronic kidney disease.

Patients with the following criteria will not be asked to participate in the study:

1. Severe untreated arterial hypertension or treated unbalanced arterial hypertension.
2. Pre-operative renal failure on dialysis.
3. Acute heart failure.
4. Acute coronary insufficiency.
5. Renal vascular surgery.
6. Cardiac surgery.
7. Permanent laparoscopy (surgery performed using a camera, with small incisions).
8. Chronic respiratory insufficiency with home oxygen therapy.
9. Acute respiratory distress syndrome.
10. A state of shock before surgery.
11. Locoregional anesthesia (spinal and epidural)
12. Refusal to participate.
13. Pregnancy.
14. Guardianship, curatorship, legal protection, or incarceration.
15. Emergency anesthesia

1. **What are the expected benefits?**

We hope to demonstrate that variations in inhaled and exhaled CO_2_ and O_2_ levels can inform the physician in real time about the balance between oxygen transport by the blood and oxygen consumption by your body. Thus, we hope that surgical management based on these variables might reduce the frequency of post-operative complications.

1. **What are the possible disadvantages?**

Participation in this study will not change the drugs that you may receive during your operation. The potential serious adverse events in the study are related to the standard administration of these drugs. We do not expect to see any additional serious adverse events.

1. **What are your rights?**

Your participation in the study will be discontinued at any time if it puts you at any risk. Furthermore, you will be free to withdraw from the study at any time without having to justify your decision. However, in that event, you will have to inform your anesthesiologist. Withdrawing from the study will not affect the quality of medical care that you will receive afterwards.

In order to help you take a decision, please be aware that you can be accompanied during the various study visits by the trusted person you have designated.

Furthermore, your participation in the study may be stopped for one of the following reasons:

- You are not following your doctor's instructions.
- You experience a serious event that may require treatment.
- The sponsor, the competent authority (the French National Healthcare Product Safety Agency, ANSM), the French Ministry of Health, or the institutional review board decides to stop the study.

As part of the clinical study in which you have been invited to participate and in order to analyze the results of the research, your personal data will be computer-processed. To this end, your personal medical data will be sent to the study sponsor or the persons or companies acting on its behalf. These data will be coded and identified by a code and your initials. Furthermore, these data may, under confidential conditions, be sent to the French health authorities.

This clinical study is being performed in the public interest and in accordance with European Union’s General Data Protection Regulation 2016/679).

Your data will be stored for up to two years after the last scientific publication related to the study. The data will then be archived (with very restricted access) for a maximum of 15 years.

In accordance with the European Union’s General Data Protection Regulation 2016/679, you have the right to access, carry, rectify, delete and limit your personal data. You also have the right to oppose the transmission of data covered by a professional duty of confidentiality and that may be used and processed in the context of this study.

These rights can be exercised through the investigating physician who is monitoring you in this study or through the data protection officer appointed by the body responsible for the processing (as defined in the General Data Protection Regulation 2016/679) and who can be contacted at the following e-mail address: dpo@chu-amiens.fr.

In the event of a dispute, you have the right to file a claim (as provided by law) with the French National Data Protection Commission (CNIL).

You can also access (directly or through the doctor of your choice) all of your medical data, as provided for in Article L1111-7 of the French Public Health Code. These rights are exercised through the doctor who is monitoring you in the study and who knows your identity.

In accordance with France’s 2012 Clinical Research Act, as amended by the government order 2016-800 dated June 16^th^, 2016 (Articles L1121-1 to L1126-6 of French Public Health Code):

- This study was approved by the “CPP Ile de France 2” institutional review board on September 14^th^, 2018, and was authorized by the French National Agency for Drug and Healthcare Product Safety (ANSM) on April 25^th^, 2018.

In accordance with the legal requirements, the study sponsor (Amiens-Picardie University Medical Center) has taken out clinical trial liability insurance cover (policy reference 147-731) with the *Société Hospitalière d'Assurances Mutuelles* insurance company (18 rue Edouard Rochet, F-69372 Lyon cedex 08, France). The policy meets the criteria defined for the protection of study participants. The insurance policy can be consulted at Amiens-Picardie University Medical Center’s Clinical Research Directorate (CHU Amiens-Picardie, F-80054 Amiens, France; tel.: +33-332-088-371), if you wish.

- - Individuals who have suffered harm as a result of their participation in clinical research may assert their rights before the regional conciliation and compensation commission for medical accidents.
  - When the study has finished, you will be personally informed by your doctor (if you so wish) of the overall results as soon as they are available.

Please keep this document. You will be able to ask questions at any time, before or during the study. Please be assured that any new information arising during your participation and that might change your decision to participate will be given to you.

After reading this study information sheet, do not hesitate to ask your doctor any questions you may have. If, after a cooling-off period, you agree to participate in this research, you must fill out and sign the participation consent form. A copy of the completed document will be given to you.

Information sheet given on : [date]...... to [participant’s name]…………………………………………,

By Dr/Professor:………………………………

**CONSENT FORM FOR PARTICIPATION IN A CLINICAL TRIAL**

| **Study title:** | | | **Individualized hemodynamic optimization guided by indirect measurement of respiratory exchange ratio in major surgery: study protocol for a randomized controlled trial (the OPHIQUE study** | | |
| --- | --- | --- | --- | --- | --- |
|  |  |  |  | | |
|  | | | |  | |
| **Sponsor**: **CHU Amiens-Picardie** | | | | **Coordinating Investigator:** | |
|  |  | 80054 Amiens cedex 1, France | | Dr. Stéphane Bar |  |
|  |  | Tel.: +33-322-088-051 | | Anesthesia and Resuscitation Department | |
|  |  | Fax.: +33-322-088-057 | | CHU Amiens-Picardie | |

F-80054 Amiens cedex 1, France

Tel.: +33-322-087-906

bar.stephane@chu-amiens.fr

I, the undersigned [family name, first name].............................................................................. born

on:…[date]……………… and residing at:……………………………………………………, certify

that I have read and understood the study information sheet given to me, and that I had enough time to think things over between being given the study information sheet and been asked to sign the consent form.

I have had the opportunity to ask Professor/Dr. [family, first name] all the questions I wanted. She/he has explained the study’s nature and objectives, together with the constraints and potential risks related to my participation in the study. I am aware that I have the right to refuse to participate in this study and that the study data may be sent to the French health authorities or legal authorities.

I am aware that I will be able to stop participating in the study at any time without having to justify my decision and that I will do my best to inform the doctor who is monitoring me in the study. I have been told that [withdrawal] will not affect the quality of my subsequent medical care.

I have been assured that decisions required for my health can be made at any time, depending on the current state of medical knowledge.

I am aware that this study was approved by the “CPP Ile de France 2” institutional review board on September 14^th^, 2018, and was authorized by the French National Agency for Drug and Healthcare Product Safety (ANSM) on April 25^th^, 2018.

The study's sponsor (Amiens-Picardie University Medical Center) has taken out clinical trial liability insurance cover (policy reference 147-731) with the *Société Hospitalière d'Assurances Mutuelles* insurance company (18 rue Edouard Rochet, F-69372 Lyon cedex 08, France).

I authorize the study personnel, persons mandated by the sponsor, and (potentially) representatives of the French health authorities to access my information under strictly confidential conditions.

I accept that the data recorded during the study may be subject to computer processing, under the sponsor’s responsibility*.*

In accordance with the provisions of the French legislation on data protection and the European Union’s General Data Protection Regulation 2016/679, I acknowledge that I have the right to access, carry, rectify, delete and limit my personal data at any time. I also have the right to oppose the transmission of data covered by a professional duty of confidentiality and that may be used and processed in the context of this study. These rights can be exercised through the investigating physician who is monitoring me in the study or through the data protection officer.

My consent in no way relieves the investigator or the study sponsor of their responsibilities to me. I retain all the rights guaranteed by law.

The overall results of the study will (if I so wish) be sent to me directly, in accordance with France’s 2002 Patient Rights and Healthcare System Quality Act.

I certify that I am affiliated to or benefit from a social security scheme.

*I ACKNOWLEDGE ALL THE INFORMATION PROVIDED IN THE STUDY INFORMATION SHEET, AGREE TO PARTICIPATE IN THE STUDY UNDER THE CONDITIONS SET OUT IN THIS DOCUMENT, AND CONFIRM THAT I HAVE RECEIVED A COPY OF THIS CONSENT FORM.*

*
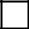
*

*The patient:*

*Family name, first name:…………………………………………………………………………*

*At [place]…………………………………………*

*Date:*

***Signature*** *(preceded by the words "Read, understood and approved")****:***

**Attestation of the investigating physician who provided the study information:**

*I have informed the above-mentioned patient about this clinical study and the related aspects. To this end, I presented the relevant documents (the study information) before he/she gave his/her consent.*

*The Investigator*

*Family name, first name:……………………………………………………………………………*

*At [place]…………………………………………*

*Date: .……………………*

***Signature:***
